# Motor Recovery After Subcortical Stroke Depends on Modulation of Extant Motor Networks

**DOI:** 10.3389/fneur.2015.00230

**Published:** 2015-11-16

**Authors:** Nikhil Sharma, Jean-Claude Baron

**Affiliations:** ^1^Stroke Research Group, Department of Clinical Neurosciences, University of Cambridge, Cambridge, UK; ^2^MRC Unit for Lifelong Health and Ageing, University College London, London, UK; ^3^The National Hospital for Neurology and Neurosurgery, London, UK; ^4^INSERM U894, Centre Hospitalier Sainte-Anne, Sorbonne Paris Cite, Paris, France

**Keywords:** motor imagery, functional imaging, fMRI, mental imagery, brain mapping

## Abstract

**Introduction:**

Stroke is the leading cause of long-term disability. Functional imaging studies report widespread changes in movement-related cortical networks after stroke. Whether these are a result of stroke-specific cognitive processes or reflect modulation of existing movement-related networks is unknown. Understanding this distinction is critical in establishing more effective restorative therapies after stroke. Using multivariate analysis (tensor-independent component analysis – TICA), we map the neural networks involved during motor imagery (MI) and executed movement (EM) in subcortical stroke patients and age-matched controls.

**Methods:**

Twenty subcortical stroke patients and 17 age-matched controls were recruited. They were screened for their ability to carry out MI (Chaotic MI Assessment). The fMRI task was a right-hand finger-thumb opposition sequence (auditory-paced 1 Hz; 2, 3, 4, 5, 2…). Two separate runs were acquired (MI and rest and EM and rest; block design). There was no distinction between groups or tasks until the last stage of analysis, which allowed TICA to identify independent components (ICs) that were common or distinct to each group or task with no prior assumptions.

**Results:**

TICA defined 28 ICs. ICs representing artifacts were excluded. ICs were only included if the subject scores were significant (for either EM or MI). Seven ICs remained that involved the primary and secondary motor networks. All ICs were shared between the stroke and age-matched controls. Five ICs were common to both tasks and three were exclusive to EM. Two ICs were related to motor recovery and one with time since stroke onset, but all were shared with age-matched controls. No IC was exclusive to stroke patients.

**Conclusion:**

We report that the cortical networks in stroke patients that relate to recovery of motor function represent modulation of existing cortical networks present in age-matched controls. The absence of cortical networks specific to stroke patients suggests that motor adaptation and other potential confounders (e.g., effort and additional muscle use) are not responsible for the changes in the cortical networks reported after stroke. This highlights that recovery of motor function after subcortical stroke involves preexisting cortical networks that could help identify more effective restorative therapies.

## Introduction

Stroke remains a leading cause of long-term disability and carries a significant social and economic cost (1, 2). After stroke, functional imaging studies of movement report widespread changes in activation of the cortical networks (3–8). The precise cognitive processes that determine these changes remain unclear. In this study, we used a data-led method to explore if the changes in movement-related networks are a result of processes specific to stroke patients (i.e., use of additional muscles) or whether they represent modulation of extent movement-related networks. Understanding this distinction in neuroplasticity is likely to help establish the driver of fMRI changes reported after stroke and help establish the most effective restorative therapies for patients (9–11).

Using a variety of tasks, numerous groups have reported changes in movement-related networks – importantly these remote changes relate to the recovery of motor performance. Movement-related fMRI activation in the ipsilesional primary motor cortex is associated with better recovery (4, 7, 8, 12, 13). Indeed it is on this model that many restorative intervention studies are based (14) changes in movement-related networks are being used to predict response to therapies (15). Yet it is possible that the changes in movement-related networks may represent an epiphenomenon of the increased difficulty involved in carrying out the task after a stroke (6).

There are several caveats when considering comparisons of patients with healthy volunteers (6). For instance, the kinematics of movements, EMG patterns, motor strategies (adaptation versus relearning), and whether movement involved different body parts in different subjects have not been monitored consistently in the MRI. In other words, it is possible that the differences reported represent a composite of cognitive processes specific to stroke patients that may not be directly related to the recovery process as such.

Understanding whether there are networks specific to stroke patients will greatly aid the understanding of the recovery process after stroke. It may allow a more targeted approach to rehabilitation as it could identify the most appropriate training programs. We explored the extent to which the widely described changes in motor networks after stroke are a result of specific processes (i.e., motor adaptation or use of different muscle) or whether they represent modulation of extant motor performance. There are two key aspects to our study.

First, to remove any biases produced by subtle differences in motor performance, we studied both motor imagery (MI) and executed movement (EM). MI is intrinsically linked to the motor system and can be used to study the motor system without actual movement (16–19). In stroke patients with normal activations during EM, we have reported abnormal hemispheric lateralization during MI that related to recovery of motor function. In other words, by studying MI as well as EM, we are able to identify aspects of task-dependent activation that relate to motor execution and those more “upstream” (20).

Second, we use a data-led approach using tensor-independent component analysis (TICA) (21). Using TICA, we examine the cortical networks that are common to stroke patients and aged-matched controls or exclusive to either. Unlike the conventional mass univariate approach, TICA is a powerful data-led approach that explores similarities as well as differences in cortical networks. Importantly, both tasks (MI and EM) from both groups (stroke and aged-matched controls) are considered the same. We are able to use a “blinded task” during the production of the independent components (ICs) as they have the same temporal profile. In other words, we make no prior assumptions as to the extent of overlap, if any, between the task-related networks in stroke patients and controls or between the MI and EM. If the widely reported changes in movement-dependent networks are related to a stroke-specific cognitive process, then this analytic approach will likely produce separate components.

We hypothesize that in recovered subcortical stroke patients, the task-related motor networks identified for both EM and MI are shared with the age-matched controls. In keeping with our reports from healthy volunteers, we expect to find networks related exclusively to EM and others that are shared with MI. Finally, we expect that in stroke patients, the task-related networks would correlate with measures of motor recovery.

## Materials and Methods

### Subjects

Twenty subcortical stroke patients were recruited (six females; mean age, 66 ± 8.8 years). Inclusion criteria were the following: (i) first-ever ischemic or hemorrhagic stroke with initial motor deficit lasting at least 2 weeks; (ii) ability to perform the motor activation task; and (iii) right-handedness. They had no past medical history of any neurological, psychiatric, or musculoskeletal disorders and were not taking regular medication. Seventeen age-matched control subjects (nine males) aged 40 years (mean, 57.6 ± 8.5 years) were recruited through local advertisement. Subjects had no history of medical disorders and were not taking regular medication. All subjects were right handed as assessed by the Edinburgh scale (22) and gave written consent in accordance with the Declaration of Helsinki, and the protocol was approved by the Cambridge Regional Ethics Committee.

All subjects underwent assessment with the Chaotic Motor Imagery Assessment (CMIA). They were excluded if unable to perform MI adequately. Chaotic Motor Imagery is defined as an inability to perform MI accurately or, if having preserved accuracy, the demonstration of temporal uncoupling (23). The full-assessment is described in detail in Ref. (24). Briefly, the assessment has three components performed in order. Where appropriate, subjects were given specific instructions to perform first-person kinesthetic MI. They were instructed not to view the scene from the third person and not to count or assign numbers or tones to each finger.

The stroke patients were assessed with the NIH Stroke Scale (NIHSS), the Action Research Arm Test (ARAT), Stroke Impact Score (SIS), and the Motricity Index. Thumb to index finger tapping over 15 s (TIT ratio) (25) and mirror synkinesia were measured. Transcranial Doppler was used to assess vasomotor reactivity and was preserved in all.

### Functional MRI

#### Motor (Imagery) Paradigm

The fMRI tasks was a block design (20, 26) of a right-hand finger-thumb opposition sequence (paced at 1 Hz; sequence 2, 3, 4, 5, 2…) and rest. There were two separate runs acquired (MI and rest and EM and rest). Subjects were instructed to keep their eyes closed throughout the session. We used bilateral fiber-optic gloves (Fifth Dimension Technologies, SA) to monitor finger movements and exclude inappropriate movement. The gloves were also used to confirm the performance of MI – after each MI block (24). Post MR subjects rated the vividness of MI performance on a seven-point scale.

#### Data Acquisition

A 3-T Brucker MRI scanner was used to acquire both T2-weighted and proton density anatomical images and T2*-weighted MRI transverse echo-planar images sensitive to the BOLD signal for fMRI (64 × 64 × 23; FOV 20 × 20 × 115; 23 slices 4 mm, TR = 1.5 s, TE 30 ms, voxel size 4 × 4 × 4).

#### Image Analysis

Analysis was carried out using TICA (21) as implemented in MELODIC (Multivariate Exploratory Linear Decomposition into Independent Components) Version 3.09, part of FSL (FMRIB’s Software Library, www.fmrib.ox.ac.uk/fsl). Only the affected hand in stroke patients was assessed. Where necessary images were flipped, the hand studied was always contralateral to the left hemisphere matching the right-hand tasks of the age-matched controls. Contralateral is therefore ipsilesional in stroke patients.

The first 12 volumes were discarded to allow for T1 equilibration effects. Preprocessing involved masking of non-brain voxels, voxel-wise de-meaning of the data, and normalization of the voxel-wise variance. Subject movement was less than 2 mm.

The preprocessed data were whitened and projected into a multidimensional subspace using probabilistic principal component analysis where the number of dimensions was estimated using the Laplace approximation to the Bayesian evidence of the model order (27). The whitened observations were decomposed into sets of vectors which describe signal variation across the temporal domain (time courses), the session/subject domain, and the spatial domain (maps) by optimizing for non-Gaussian spatial source distributions using a fixed-point iteration technique (28). Estimated component maps were divided by the standard deviation of the residual noise and thresholded by fitting a mixture model to the histogram of intensity values. The time course of each IC was then entered into a general linear model of the convolved block design of Task versus Rest.

An IC was considered to be involved in MI or EM if a one-way *t*-test found the subject scores to be significantly different from zero across subjects. When an IC was significantly involved in both tasks, then a paired *t*-test (*p* < 0.05 corrected for multiple comparisons) was performed on the subject score for each task. In the stroke group, the subject scores of each remaining component were correlated (Spearman *p* < 0.05 corrected for multiple comparisons) with the impairment scores.

## Results

### Behavioral Results

Four control subjects and eight stroke patients were excluded because of chaotic motor imagery. Twelve stroke patients remained [eight left hemisphere; four females; for full demographic details see Sharma et al. (24)]. There was no difference in score between the stroke group and control subjects.

All subjects suppressed movement and all were compliant during the fMRI task. Median post-MRI MI vividness score was 6 (range, 4–7).

### fMRI Data

No distinction was made between tasks until the final stage of processing. As 25 subjects performed two tasks, MI and EM, 50 “blinded” tasks were processed. As no distinction was made between imagery and EM during the generation of the ICs, we use the term “blinded.”

A subject score for each IC is produced that includes the effect size for the 50 blinded tasks (13 controls subjects, EM and MI, 12 stroke patients) for the associated spatiotemporal process shown in the spatial map.

Twenty-eight ICs were defined by TICA. ICs that identified artifact recognized by previously published patterns and high frequency were excluded by visual inspection. ICs driven by outliers or were not significant across either task were also excluded. Therefore, only components in which the subject scores were significantly different from zero (for either the stroke or control group for either task) were included.

Seven ICs remained. Each component was significantly involved in both the stroke group and the control group. As hypothesized, some ICs were shared between EM and MI (subject scores significantly greater than zero for both tasks in both groups) and some were exclusive to EM (subject score greater than zero for EM only in both groups).

**Figure 1:**
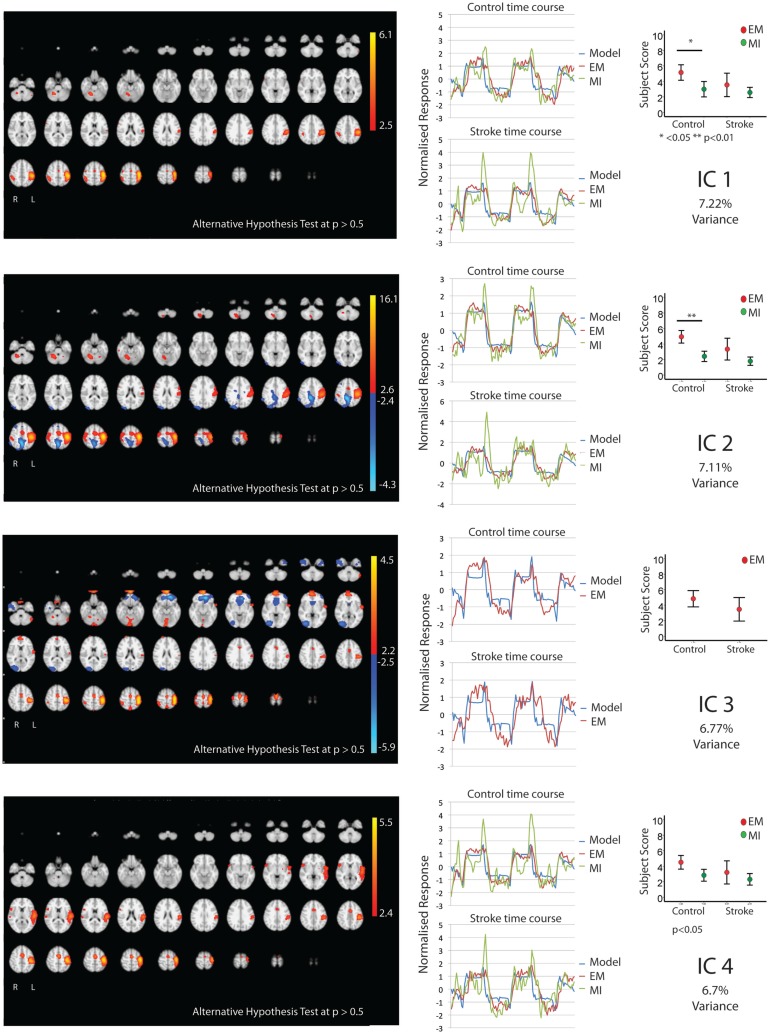
**The figures show the involvement of each IC across the whole brain with a standard threshold of *p* > 0.5 (alternative hypothesis test) and the variance it accounts for out of the total explained variance**. In four stroke patients, the images were flipped so that the left hemisphere is always contralateral to executed movement/motor imagery. The left hemisphere equates to the ipsilesional hemisphere. The scales show the transformed *z*-score, orange is activation, and blue is deactivation. The normalized time course response is shown for each task and the full model fit (full model fit = blue, executed movement = red, and motor imagery = green). The mean subject scores with standard error bars are shown for each task and differences highlighted (executed movement = red, motor imagery = green). The time course and subject score for each task are shown.

**Figure 2:**
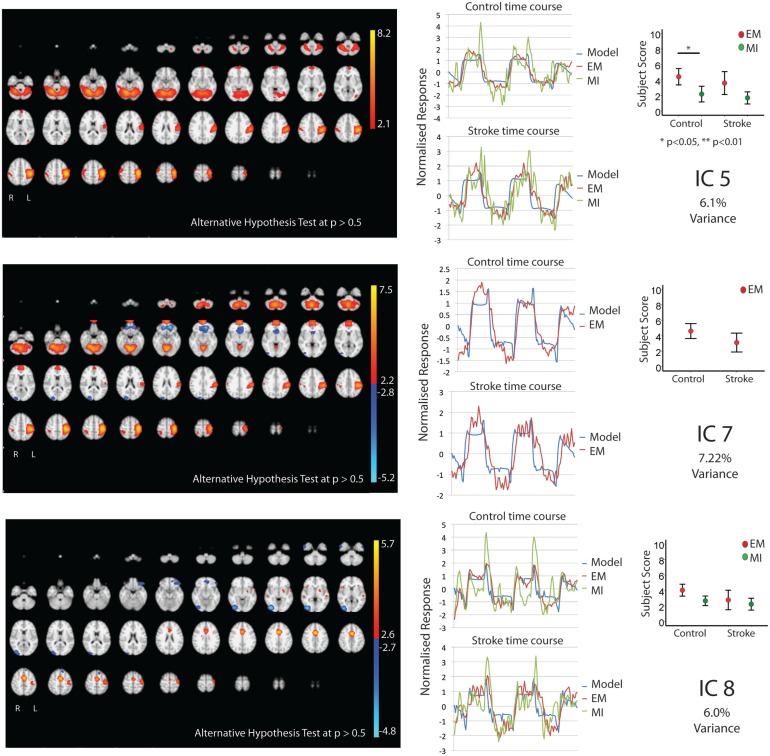
**The figures show the involvement of each IC across the whole brain with a standard threshold of *p* > 0.5 (alternative hypothesis test) and the variance it accounts for out of the total explained variance**. In four stroke patients, the images were flipped so that the left hemisphere is always contralateral to executed movement/motor imagery. The left hemisphere equates to the ipsilesional hemisphere. The scales show the transformed *z*-score, orange is activation, and blue is deactivation. The normalized time course response is shown for each task and the full model fit (full model fit = blue, executed movement = red, and motor imagery = green). The mean subject scores with standard error bars are shown for each task and differences highlighted (executed movement = red, motor imagery = green). The time course and subject score for each task are shown.

Figures 1 and 2 show the whole brain activations and deactivations, the time course (BOLD), subject scores, and percentage of total variance explained. Table 1 summarizes the areas involved [labeled using the Jülich Atlas (29)].

**Table 1:** **Regions activated or deactivated in each independent component**.

	Activated in both executed movement and motor imagery										Executed movement only			
	IC1		IC2		IC4		IC5		IC8		IC3		IC7	
	Left	Right	Left	Right	Left	Right	Left	Right	Left	Right	Left	Right	Left	Right
BA44					↑									
BA4	↑		↑^a^	↓	↑		↑		↑		↑		↑	
Pre-SMA														
SMA	↑				↑				↑	↑	↑	↑		
PMd	↑	↑	↑	↑	↑			↑	↑		↑	↑	↑	
Area 1	↑	↑	↑	↑	↑		↑	↑			↑	↑	↑	
Area 2	↑	↑	↑	↑	↑		↑	↑	↑		↑	↑		↑
3a			↑		↑				↑		↑		↑	
3b	↑		↑	↑	↑		↑		↑		↑	↑	↑	↑
hIP1		↑												
hIP2	↑	↑	↑	↑			↑					↑		↑
hIP3	↑	↑						↑					↑	
SPL(7A)													↑	
SPL(7PC)	↑	↑	↑		↑		↑				↑	↑		
IPC(PFop)	↑		↑		↑						↑		↑	↑
lPC(PFt)		↑		↑				↑			↑			↑
IPC(PFm)				↓										
IPC(Pga)														
IPC(PF)														
Thal_premotor														
Thal_motor														
Thal_Somatosensory														
Caudate														
TE														
CB		↑		↑			↑	↑			↑	↑	↑	↑

*^a^Small area of deactivation in a more dorsal area*.

### Independent Components (IC 1, 2, 4, 5, 8) Shared by Executed Movement and Motor Imagery

Five components (IC 1, 2, 4, 5, 8; Figures 1 and 2) were significantly involved in both age-matched controls and stroke patients and were common to both EM and MI (subject scores > 0 for both tasks in both groups). Together these five ICs explained 33% of the total explained variance. All of the components significantly correlated with the active blocks of the task.

In three of the components (IC1, 2, 5), the subjects score was greater during EM than during MI in the age-matched controls only – no such difference was found in the stroke group. IC1 involved activation of the contralateral motor areas and bilateral involvement of premotor and parietal areas. More specifically, there was contralateral activation of BA4a, SMA, BA3b, and parietal areas [IPC(PFo)]. There was bilateral activation of PMd, both SI and SII, and parietal areas (hIP2,3 and 7PC). There was ipsilateral activation of the parietal areas [hIP1, IPC (Pft)] and cerebellum.

Similarly IC 2 predominantly showed contralateral activation of BA4, parietal lobe [IPC (Pfo)], and bilateral activation of PMd, SI, SII, parietal lobe (hIP2), and contralateral cerebellum. However, in a different topographical location (more dorsal), there was a small degree of deactivation of the contralateral BA4a and ipsilateral parietal lobe [IPC (Pfm)].

Independent component 4 was exclusively contralateral. While sensory motor areas (BA4, SMA, PMd, SI, SII, BA3a,3b) and parietal areas [both SPL(7PC) & IPC(Pfop)] were involved, it was the only IC to involve BA44. Notably there was no cerebellar activation.

Independent component 5 shared many features of IC1 and IC2, with involvement of primary and secondary motor areas as well as parietal areas. More specifically, there was contralateral activation of BA4, BA3b, parietal areas [SPL(7PC)], bilateral activation of SI, SII, and cerebellum, and ipsilateral parietal areas [IPC(PFt)]. Notably, it was the only component with only ipsilateral involvement of PMd and parietal area (hIP3).

Independent component 8 was similar to IC4 with predominantly contralateral activation (except for SMA). This involved BA4a, BA3a. In contrast, it was the only component with contralateral PMd, SII activation.

### Independent Components Involved During Executed Movement Only (IC 3, 7)

Two components, IC 3 and 7, were involved during EM only explaining 6.77 and 7.22% of total variance, respectively. IC3 involved activation of the contralateral BA4, BA3a, and IPC, with bilateral activation of SMA, PMd, S1&2, BA3b, parietal area (SPL), and cerebellum. There was ipsilateral activation of parietal area (hIP2). IC7 activated the contralateral BA4, PMd, S1, BA3a, and parietal areas [HIP3 SPL (7A)], with bilateral involvement of BA3b, parietal area [IPC (PFop)], and cerebellum. There was ipsilateral activation of SII, hIP2, and parietal area [IPC (Pft)].

### Relationship of Motor Imagery and Executed Movement ICs in Stroke Patients to Motor Performance and Time Since Stroke (IC 1, 3, 7)

In the stroke group, there were two ICs (1 and 3) that related to motor performance. While IC 3 was exclusive to EM, it is notable that IC1 – a component common to both EM and MI – is also related to motor performance.

As there was no significant difference between the IC1 subject scores for each task, both tasks were explored together. There was a significant positive correlation between this combined IC1 subject score and the Motricity (Arm) scores (ρ = 0.581; *p* < 0.05), i.e., the greater the activity within this network the better the recovery. The same overall pattern of correlation was mirrored with SIS (ρ = 0.501; *p* < 0.05) and Motor Activity Log (ρ = 0.540; *p* < 0.05).

Independent component 3 (EM only) was positively correlated with SIS (ρ = 0.648; *p* < 0.05). In other words, greater activation of IC3 was associated with better recovery.

Finally, IC7 was negatively correlated with time since stroke (ρ = 0.592; *p* < 0.05), i.e., this activation within this network reduced with time since stroke.

**Figure 3:**
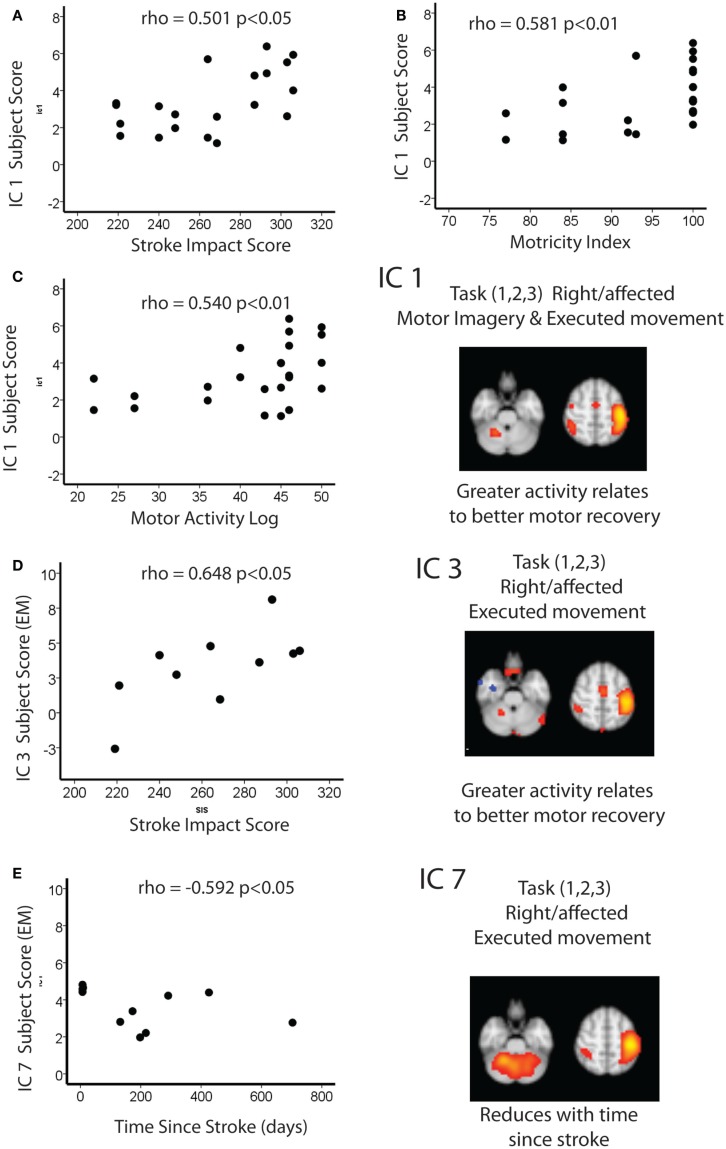
Spearman correlations between IC1 and **(A)** stroke impact score **(B)** motricity index and **(C)** motor activity log. Spearman correlations between IC3 and **(D)** stroke impact score and **(E)** time since stroke.

Figure 3 summarizes these findings.

## Discussion

We report that the cortical networks that relate to recovery of function are not specific to stroke but instead represent modulation of existing networks. As expected, most cortical networks were shared between EM and MI (accounting 33.13% of the total explained variance), with only two networks that were exclusively found during EM (accounting for 13.99% of the explained variance). The absence of any cortical networks specific to stroke patients suggests that the changes in cortical networks reported after stroke are not a result of a subtle biases exclusive to stroke patients – this may have included a motor behavior like adaptation (adjusting movement to new demands) or other potential confounders such as effort or attention. This work emphasizes that recovery of motor function involves preexisting cortical networks that may help identify more effective restorative therapies for stroke patients.

This study further extends the close similarities between MI and EM. We report that the first IC (IC1 accounting for 7.22% of the total explained variance) was involved in both groups and in both tasks (EM and MI). It involved activation of the contralateral motor areas and bilateral involvement of premotor and parietal areas. The involvement of the motor cortex – an area pivotal to motor learning (30) – strengthens the rationale for using MI training after stroke. We found that greater involvement of IC1 was associated with better recovery of motor performance after stroke. As this IC is shared between tasks, it suggests that a key aspect of the recovery process occurs “upstream” from motor execution. Importantly, this network is shared with age-matched controls, implying that it is not exclusive to stroke.

Consistent with our previous findings in healthy volunteers (31), we report two networks that are exclusive to motor execution (IC 3 and 7 explaining 6.77 and 7.22% of total variance, respectively). The areas common to both are the contralateral primary and secondary motor areas (although IC7 was largely bilateral with marked cerebellar involvement). This is likely explained by the differences between EM and imagery. First, EM involves discharge via the corticospinal tract (CST) that we have previously suggested dominates the movement-related activation (6). Second, the resultant movement produces afferent sensory feedback to the motor system.

We postulate that the IC3 is responsible for the discharge via the CST, given the near exclusive activation of the primary motor cortex. In support of this view, greater subject score of this network is associated with better recovery of motor performance (as assessed with the SIS). This is consistent with the findings from transcranial magnetic stimulation (TMS) studies that suggest that preservation of the CST is associated with a better recovery of motor performance after stroke (32–34).

It is likely that IC7 is related to the sensory feedback during motor execution, given the significant bilateral cerebellar activation. We found that this network reduces with time since stroke, similar to other reports that use network analysis of resting-state fMRI (35). Remarkably, both of these movement-related networks are shared with age-matched controls, again consistent with the idea that recovery of motor performance after subcortical stroke involves modulation of extant networks rather than stroke-specific networks.

The interactions between the primary motor cortices are the foundation for numerous interventions after stroke (4, 14, 15, 25). These interventions can include but are not limited to TMS [see Cramer et al. (7) for an overview]. Overall, there is growing support for this model (13, 36). In addition to the contralateral motor cortex activation, we identify an area of deactivation within the more dorsal aspect of the ipsilateral/contralateral motor cortex (IC2). While there are complex interactions between the motor cortices during movement, the topographical distributions of these areas, i.e., away from the “hand area” make interpretation difficult. Of course, the model previously suggested (4, 14, 15, 25) is an oversimplification and fails to capture the existence of multiple cortical networks that are involved in the recovery process. It may also apply to certain stages and degrees of recovery only. Importantly, future work needs to address the effect of interventions like TMS and tDCS on multiple cortical networks (37, 38) as their effects may be more nuanced that simply increases or decreases activation. This highlights the importance of selecting the most appropriate training that should be combined with TMS or tDCS (39).

This study has a number of limitations. The patients included were relatively well recovered and whether similar results would be found in a more severely affected group is unknown. We studied only subcortical stroke. It is feasible that our findings may not apply to cortical strokes. We studied both right- and left-hemisphere strokes in right handers and flipped the MR images to one side in order to carry out the TICA on a meaningful sample size. Again, we cannot rule out that findings for dominant and non-dominant hemisphere stroke may differ. We excluded stroke patients who were performing chaotic motor imagery, and it is therefore possible that these patients may have used alternative cognitive processes that could have been interpreted as being stroke specific – though one would not expect these networks to relate to the recovery of motor performance as such. Although TICA can examine cortical networks that are shared between tasks, it has limitations (40). By considering EM and MI together in TICA analysis, we must assume that the tasks have the same temporal profile. It is entirely possible that this approach has overlooked cortical networks that have different temporal profiles – this limits the use of TICA-based fMRI as a biomarker for patient selection. However, if that was the case, then one would expect those areas to have been highlighted by earlier mass univariate fMRI studies.

## Conclusion

In summary, we find that in our sample of well-recovered subcortical stroke patients, cortical networks associated with recovery of motor performance include some cognitive processes upstream from actual movement while others are exclusively dependent on execution. Importantly, all of these networks were present in age-matched controls, suggesting that recovery of motor performance after stroke requires existing cortical motor networks rather than recruiting additional areas. These results also imply that the models of motor recovery after stroke [suggested by Ward and Cohen (14)] should be updated to consider movement as a combination of distinct cortical networks, each of which may have a separate contribution to recovery. Finally, we need to explore how each of these networks is affected by non-invasive stimulation to fully exploit their therapeutic potential.

## Conflict of Interest Statement

The authors declare that the research was conducted in the absence of any commercial or financial relationships that could be construed as a potential conflict of interest. The Review Editor Andreas Charidimou declares that, despite being previously affiliated to the same institution as Nikhil Sharma, and despite having collaborated on publications (not related to the current topic) in the last 2 years with Jean-Claude Baron, the review process was handled objectively.
